# Dementia and Its Profound Impact on Family Members and Partners: A Large UK Cross-Sectional Study

**DOI:** 10.1097/WAD.0000000000000647

**Published:** 2024-11-07

**Authors:** R. Shah, M.S. Salek, F.M. Ali, S.J. Nixon, K. Otwombe, J.R. Ingram, A.Y. Finlay

**Affiliations:** * Division of Infection and Immunity, School of Medicine, Cardiff University; ‡ Institute of Medicines Development; § Multiple Sclerosis Society, Cardiff; † School of Life & Medical Sciences, University of Hertfordshire, Hatfield, UK; ∥ Statistics and Data Management Centre, Perinatal HIV Research Unit, Chris Hani Baragwanath Academic Hospital, University of the Witwatersrand, Johannesburg, South Africa

**Keywords:** family impact, dementia, Alzheimer disease, Family-Reported Outcome Measure-16, FROM-16, family members, partners, quality of life

## Abstract

**Introduction:**

Dementia can adversely affect the quality of life (QoL) of family members/partners of those affected. Measuring this often-neglected burden is critical to planning and providing appropriate support services. This study measures this impact using the Family-Reported Outcome Measure (FROM-16).

**Methods:**

A large UK cross-sectional online study through patient research platforms, recruited family members/partners of people with dementia, to complete the FROM-16.

**Results:**

Totally, 711 family members/partners (mean age=58.7 y, SD=12.5; females=81.3%) of patients (mean age=81.6, SD=9.6; females=66.9) with dementia completed the FROM-16. The FROM-16 mean total score was 17.5 (SD=6.8), meaning “a very large effect” on QoL of family members, with females being more adversely impacted.

**Conclusions:**

Dementia profoundly impacts the QoL of family members/partners of patients. Routine use of FROM-16 could signpost provision of care support, reducing family members’ burnout. Such routine data could be used in economic analysis of the burden of dementia as well as in predicting institutionalization.

Dementia has become one of the greatest healthcare challenges of the 21st century due to the resulting high need for medical, social, and institutional care.^1,2^ Globally, an estimated 52 million people are living with dementia and this number will double by 2030 and triple by 2050.^3^ In the UK there are an estimated 900,000 people living with dementia and it is predicted that this number may rise to 1.6 million by 2050.^4^ The most common form of dementia is Alzheimer disease (AD), accounting for 50% to 70% of dementia cases.^2^ Family members/informal carers serve as a major source of care for people with dementia across the world.^5,6^ In the UK, two-thirds of people with dementia live at home, receiving most of their support from family carers.^7^ As dementia is characterized by ongoing decline of brain functioning manifested by memory loss, disturbances in language and cognitive functions, changes in behaviors, and impairments in activities of daily living,^6,8^ it has a proven impact on QoL of family carers.^9^ Family members may feel physically and emotionally drained dealing with the practical challenges of caring for a relative with dementia leading to major life changing circumstances.^10^ Compared with other health conditions, the impact of person’s dementia may be more debilitating as families may struggle to connect with their loved ones due to changes in their personalities and behaviors. This study aims to measure the QoL impact of having a relative with dementia and AD on family members/partners using a family-specific generic measure, the Family Reported Outcome Measure (FROM-16).

## METHODS

### Study Design and Participant Recruitment

The data used in this study came from a large online cross-sectional study of family members/partners of people with a wide range of medical conditions. In this study, family members/partners of people with dementia and AD were recruited online through Join Dementia Research (JDR) and Healthwise Wales (HWW).^11^

### Ethical Considerations

Ethical approval was given by the Cardiff University School of Medicine Research Ethics Committee (SREC reference: 21/19), which conforms to the principles embodied in the Declaration of Helsinki. Convenience sampling was used for recruitment of the study participants. The study was open to UK family members/partners of patients, aged 18 years and older and capable of using an electronic device. The exclusion criteria included family members of deceased patients, those aged below 18 years, not capable of using electronic devices and family members not living in the UK. The family members/partners chose whether to participate in the study after reading the participant information sheet embedded in the online questionnaire. All family members who participated in the study provided consent, electronically.

### Measurement of Family QoL

The impact on family members/partners was measured using the FROM-16, a generic family QoL instrument, which measures the impact of any disease on the QoL of adult family members or partners of patients of any age.^12^ The FROM-16 was created following interviews with 133 family members of patients across 26 medical specialities, exploring in depth the impact of a relative’s health condition on family members.^13^ The FROM-16 comprises 16 items, each with three response options: “Not at All” (score=0), “A Little” (=1) and “A Lot” (=2). The 16 items are divided into 2 categories (domains): emotional (comprising 6 items, maximum score of 12) and personal and social life (comprising ten items, maximum score of 20).^12^ Although FROM-16 has 2 distinct domains, FROM-16 scores are calculated as a total summary score. The lowest possible score of FROM-16 is 0, and the highest 32. The higher the total score, the greater is the negative effect on the family member’s QoL. The interpretation of scores is described using validated score meaning bands.^14^ The FROM-16 has demonstrated high internal consistency (n=120, Cronbach α=0.91) and high reproducibility (n=51, ICC=0.93), with a mean completion time of 2 minutes. Construct validity was proven through the correlation between FROM-16 and WHOQOL-BREF total scores (n=119, *r*=−0.55, *P*<0.001), and the correlation between FROM-16 and the patient’s overall health score (n=120, *r*=−0.51, *P*<0.001).^13^ Responsiveness to change has been established and the Minimal Important Change (MIC) score value of FROM-16 is 4.^15^ The FROM-16 has been mapped to EQ-5D-3L^16^ and could potentially be used to convert QoL scores into utility values, allowing inclusion of disease impact on family members in health economic analysis.

### Procedure

The online study was carried out using the Jisc academic survey platform, which is general data protection regulation compliant. The online study questionnaire had two sections; section one asked family members/partners to complete some basic information about their family members with dementia (sex, age, occupation, health condition, and country of residence). Section two asked family members/partners to answer some basic demographic questions about themselves (sex, age, occupation, and relationship to patient) and complete the FROM-16 questionnaire.

Patient and public involvement: Two patients and one family member were involved in the study as research partners. They were actively involved at all stages of the study design, participated in research team meetings, and reviewed all study materials.

### Data Analysis

Descriptive analysis was carried out and included calculating the mean, median, SD, and interquartile range of quantitative variables and frequency and proportion for categorical variables. Independent samples *t-test*/Mann-Whitney *U* test and ANOVA were used to compare between groups. Age was categorized into four groups: 18 to 29, 30 to 59, 60 to 79, and 80 to 91 years based on median (two below and two above median). Descriptive banding was assigned to the FROM-16 scores to describe severity of the impact on family members/partners. Data were analyzed using IBM SPSS Statistics for Windows, version 27.

## RESULTS

### Sociodemographic Characteristics of the Study Participants

A total of 711 family members/partners (mean age=58.7 y, SD=12.5; females=81.3%) of patients (mean age=81.6, SD=9.6; females=66.9%) completed the FROM-16 (Table 1), who described their relative’s condition as either “dementia” or “Alzheimer disease”. The family members/partners were mostly from England (67.9%), followed by Wales (24.5%), with 40.4% retired, 39% in paid jobs and 10.7% in part-time employment (Table 1). Family members were mostly sons/daughters of the patients (63.4%) followed by spouses/partners (21.9%) (Table 1).

**TABLE 1:** Descriptive and Sociodemographic Characteristics

Characteristics	Mean (SD) or N (%)
Family member age (y)	
Mean (SD)	58.7 (12.5)
Median	60
Range (IQR)	18-91 (15)
Family member gender	
Male	132 (18.6)
Female	578 (81.3)
Prefer not to say	1 (0.1)
Family member occupation	
In paid work	277 (39)
Part-time job	76 (10.7)
Unemployed	18 (2.5)
In unpaid work	15 (2.1)
Education/training	11 (1.5)
Homemaker	22 (3.1)
Retired	287 (40.4)
Rather not say	5 (0.7)
Family member’s relationship	
Spouse/partner	156 (21.9)
Son/daughter	451 (63.4)
Parent	13 (1.8)
Sibling	22 (3.1)
Other (father/mother in law, grandparent, uncle/aunt, grandson/granddaughter, brother/sister in law, nephew/niece, cousin, friend)	69 (9.7)
Patient age (y)	
Mean (SD)	81.6 (9.6)
Median	83
Range (IQR)	18-100 (12)
Patient gender	
Male	235 (33.1)
Female	476 (66.9)
Prefer not to say	
Health conditions (self-reported)	
Alzheimer disease	345 (48.5)
Dementia	366 (51.5)
Patient occupation	
In paid work	6 (0.8)
Part-time job	0
Unemployed	3 (0.4)
In unpaid work	6 (0.8)
Education/training	1 (0.1)
Homemaker	4 (0.6)
Retired	696 (97.9)
Rather not say	1 (0.1)
Country of residence in the UK	
England	483 (67.9)
Northern Ireland	6 (0.8)
Scotland	48 (6.8)
Wales	174 (24.5)

### FROM-16 Scores

The FROM-16 mean total score was 17.48 (SD=6.8, median=17, IQR=9), with a mean score for the emotional domain=7.86 (SD= 2.6) and for the personal and social life domain=9.6 (SD=5.1). As for the individual FROM-16 items, “feeling sad” had the highest mean score of 1.65 (SD=0.53), followed by “feeling worried” (mean=1.59, SD=0.55), “difficulty caring” (mean=1.48, SD=0.60), and “feeling frustrated” (mean=1.41, SD=0.63). The “effect on family activities,” “effect on holidays,” “effect on sleep,” “time for self,” and “family relationships” had the next highest mean scores (Table 2).

**TABLE 2:** Mean FROM-16 Scores and Individual Item Scores of Family Members/Partners of People With Dementia (n=711)

FROM-16	Description	Mean (SD)	Range
Total FROM-16 mean score	Overall	17.48 (6.8)	0-32
Domain score	Emotional domain	7.86 (2.6)	0-12
	Personal and social life domain	9.62 (5.1)	0-20
FROM-16 individual Items score	Worried	1.59 (0.55)	0-2
	Angry	0.86 (0.74)	0-2
	Sad	1.65 (0.53)	0-2
	Frustrated	1.41 (0.63)	0-2
	Talking about thoughts	0.87 (0.77)	0-2
	Difficulty caring	1.48 (0.60)	0-2
	Time for self	1.14 (0.72)	0-2
	Everyday travel	0.71 (0.79)	0-2
	Eating habits	0.57 (0.73)	0-2
	Family activities	1.27 (0.66)	0-2
	Holiday	1.23 (0.78)	0-2
	Sex life	0.66 (0.83)	0-2
	Work or study	0.78 (0.75)	0-2
	Family relationships	1.10 (0.71)	0-2
	Family expenses	0.98 (0.77)	0-2
	Sleep	1.18 (0.72)	0-2

### Comparison of Family Members/Partner Quality of Life for Sex and Age

There was a significant difference in FROM-16 mean total scores between male (mean score=15.71, SD=6.96, range=0 to 30, n=132) and female family members (mean=17.88, SD=6.70, range=1 to 32; n=578, *P*=0.001), with females having experienced significantly more impact across “sadness,” “frustration,” “difficulty caring,” “effect on family relationships,” and “sleep” due to their relative’s dementia (*P*<0.05) (Table S1, Supplemental Digital Content 1, http://links.lww.com/WAD/A510). Male family members/partners reported significantly more impact on their sex life than female family members/partners (*P*=0.01).

There was a small difference in the FROM-16 scores across age groups (18 to 29; 30 to 59; 60 to 79; 80 to 91) (Table S2, Supplemental Digital Content 1, http://links.lww.com/WAD/A510); however, this was not significant (Table S3a, Supplemental Digital Content 1, http://links.lww.com/WAD/A510). Although ANOVA showed some difference between the age groups for emotional impact, this was not significant in post hoc analysis (Table S3a and b, Supplemental Digital Content 1, http://links.lww.com/WAD/A510).

**FIGURE 1:**
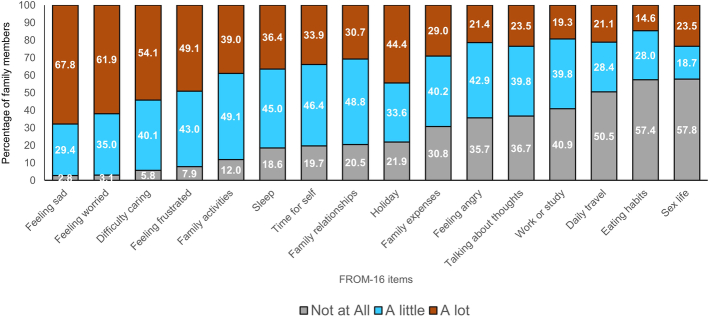
Impact of relative’s dementia and Alzheimer disease on family members/partners across 16 items of FROM-16.

The QoL impacts most reported by family members/partners were feeling sad (97.2%; a little=29.4%, a lot=67.8%), feeling worried (96.9%; little=35%, lot=61.9%), difficulty caring (94.2%; little=40.1%, lot=54.1%), feeling frustrated (92.1%; little=43%, lot=49.1%), impact on family activities (88.1%; little=49.1%, lot=39%), impact on sleep (81.4%; little=45%, lot=36.4%), and effect on family relationships (79.5%; little=48.8%, lot=30.7%) (Fig. 1).

### Impact of Person Dementia Across Relationships

The FROM-16 mean total score differed depending on the relationship of the family member to the patient. The QoL of spouses/partners (n=156) of people with dementia and AD was more impacted (mean FROM-16 score=19.46, median=20, SD=7.3, range: 0 to 32, IQR=9) than that of sons/daughters (mean=17.24, median=16, range: 1 to 32, IQR=9, n=451), siblings (mean=13.55, median=14, range=3 to 26, IQR=10.75, n=22), and other relatives (mean=15.65, median=16, range=1 to 30, IQR=11, n=69;) (*P*<0.05) (Table S4, Supplemental Digital Content 1, http://links.lww.com/WAD/A510, Table S5a-b, Supplemental Digital Content 1, http://links.lww.com/WAD/A510).

### Contextualizing the Study Participants’ Quality of Life Using the FROM-16 Severity Score Bands

The degree of severity of impact experienced by the family members/partners was explored using the FROM-16 score meaning descriptor bands:^14^ 53% had a mean FROM-16 score ≥17, indicating “a very large effect” on the QoL of these family members. Only 0.4% of family members experienced “no effect” of their relative’s dementia on their QoL (Table 3).

**TABLE 3:** FROM-16 Score Banding Describing Impact on Quality of Life of Family Members/Partners (n=711)^14^

FROM-16 score banding	Number of family members (percentage of family members)
No effect (0-1)	3 (0.4)
A small effect (2-8)	65 (9.1)
A moderate effect (9-16)	266 (37.4)
A very large effect (17-25)	270 (38.0)
An extremely large effect (26-32)	107 (15.0)
Total	711 (100.0)

## DISCUSSION

The purpose of this research was to explore the impact of a person’s dementia on the QoL of their family members/partners using a family-specific generic measure, the FROM-16. Given that most dementia carers are family members,^6^ the FROM-16 is an appropriate tool for measuring family caregiving impact, as it considers all aspects of disease impact on families.

The findings of this study suggest that family members/partners living with and caring for a relative with dementia experience a very large impact on their QoL. This is consistent with other studies.^17–19^ In our study, caring for a relative with dementia greatly impacted family members’/partners’ emotional health, with most feeling sad, worried, and frustrated. These findings are consistent with another UK study in which 82% of carers reported medium (64%) to high (18%) levels of anticipatory grief^20^ and with an Italian study,^21^ where dementia carers with high-burden experienced depressive symptoms. Feelings of sadness and frustration are not surprising as it may be very difficult and painful for family members/partners to deal with the fact that they are no longer recognized by their relative with dementia.^2^ Poor emotional health can lead to anxiety and depression.^17^ A study from South Korea^22^ showed a clear association between dementia caregiving and increased risk of depression. Carers with depression have higher ratings of burden^9^ and suicidal ideation.^22,23^ Depression in dementia carers has been associated with the patient’s neuropsychological symptoms, being female, and being a spouse caregiver.^17,18^

In our study, 94.2% of family members and partners reported difficulty caring for their relative with dementia. This is not unsurprising given the highly demanding nature of dementia caregiving,^19,23^ which could result in spillover effects on family activities and relationships.^23,24^ In our study, 88.1% of family members/partners reported effects on family activities, while 79.5% reported caring for their relative with dementia had adverse effects on family relationships, with females being affected significantly more than males. As females often play a central role in running the household, caring for a relative/spouse with dementia might not allow them to attend to other family members.

In our study, 81.4% of family members/partners reported that their relatives’s dementia affected their sleep, with female carers experiencing significantly more sleep disturbance than males. Sleep disturbance can negatively affect QoL^25^ and may contribute to immune suppression.^26^ Many studies have reported that sleep disruption and sleep quality are impacted in dementia carers.^17^ Sleep disruption leads to poor sleep quality and higher rates of daytime sleepiness among carers of people with dementia compared with noncarers.^2^ Sleep quality of family members of dementia patients may be affected by the type of dementia: for example, spouses of people with frontotemporal dementia had lower ratings of sleep quality and higher use of medications to aid sleep than spouses of people with semantic dementia.^27^ Another study reported that caregiver sleep was impacted irrespective of whether or not living with a person with dementia, suggesting that stress and worries about a loved one with dementia can lead to sleeplessness in family members whether or not directly involved in caring.^28^ This underscores the relevance of a family member/partner specific QoL instrument that would capture such aspects, regardless of whether the family member/partner was living with the relative.

In our study, 80.3% of family members reported that their relative's dementia impacted time for self. This is consistent with the findings from other studies.^2,10,29,30^ Caring for their relative with dementia becomes a priority for family members/partners, often leading to the postponement of their own medical care, leading to greater impairment of their health and QoL^2^ impacting carers’ personal lives, difficulties in maintaining their professional and social life and affecting their parental responsibilities.^19^ In our study, 78% of family members/partners of people with dementia reported an impact on their holidays. Although the high caregiver burden and practical issues may not allow time for family holidays, this study was conducted during the COVID-19 pandemic, which impacted everyone’s holiday plans.

Dementia carers with financial concerns have been shown to have poor mental health and QoL.^2,5,31^ In our study, 69.2% of family members reported that their relative’s dementia led to increased family expenses. This may be due to family members/partners having to leave their jobs or go part-time in order to care for the person with dementia.^32^ In the UK, the caregiver’s allowance^33^ may not be sufficient for families to meet the extra caring expenses, suggesting that more needs to be done to support such families, especially in consideration of the substantial savings that family/informal care provides to the public healthcare service.^34^

When considering the extent of the impact of a person’s dementia on different relatives, our study suggests that spouses/partners were most impacted, followed by parents and adult children. Although spouses/partners, adult children and parents had an overall FROM-16 score >17, suggesting “a very large effect on QoL,” there was a significant difference in the impact between spouses/partners compared with that of other relationships and females were significantly more impacted than males, except for sex life.

There was no significant difference in FROM-16 scores between the four age groups (ie, 18 to 29; 30 to 59; 60 to 79; 80 to 91). Although the age group (30 to 59) years was emotionally more impacted than other age groups, this difference was not significant. This is in contrast to findings from other studies,^31,35^ which suggest that younger family members are more burdened and experience depressive and anxious symptoms.

In our study, 53% of family members experienced a “very high” to “extremely high” impact (FROM-16 score ≥17) on their QoL. This is comparable to the FROM-16 scores reported by family members of people with neurological conditions (55% with FROM-16 score ≥17; n=1620) but higher than that reported by the family members of people with oncological conditions, (40% with FROM-16 scores ≥17 n=241) and psychiatric conditions (48% with FROM-16 score≥17; n=325) in a study conducted with family members of patients across 27 medical specialities.^33^

Comparing the findings of this study with other FROM-16 studies across other conditions suggests that dementia has a greater impact on family members than many other conditions. A global study on family impact of COVID-19 survivors had a mean FROM-16 score of 15.0,^36^ suggesting that dementia carers experienced much greater impairment on their QoL compared with family members of COVID-19 survivors. A global study on myalgic encephalomyelitis/chronic fatigue syndrome (ME/CFS) during the COVID-19 pandemic reported a mean FROM-16 score of 17.9,^37^ suggesting a comparable family impact to our study. However, FROM-16 scores of other studies conducted before the COVID-19 pandemic are much lower. For example, a general study on the impact on family members of patients with chronic disease across 26 medical specialities had a mean FROM-16 score of 12.4.^38^ Another study on the impact on family members/partners of oncology patients reported a mean FROM-16 score of 11.8.^39^

### Study Limitations

Our study has some limitations. First, this study was conducted after the second wave of COVID-19 in the UK, indicating that the impact of dementia on family members/partners could have been exaggerated. However, research suggests that family carers and their relatives living with dementia experienced challenges to in-home care before the COVID-19 pandemic.^40^ Second, selection bias is possible as this study was conducted online with family members registered with JDR and HWW. Third, the study did not ask questions on ethnicity, so we cannot comment on the diversity of the sample.

### Implication for Practice

Family members/partners of people with dementia experience a very large negative impact on their QoL, limiting their personal, social, and emotional well-being, which is often ignored and excluded from healthcare policy, planning, and economic appraisal. Our results are consistent with previous findings^2,11,14,15,18,21,25^ related to the family burden of dementia, demanding action that would support family-centred care. Research has demonstrated that poor QoL in family members of people with dementia is a predictor of nursing home (long-term care facility) placement for dementia patients,^41,42^ suggesting that increased prevalence of dementia and poor QoL in family carers would trigger a consequential rise in healthcare costs.^5^ It is not a common practice to conduct structured assessments of the needs of the patient’s family members or partners,^2^ compromising not only the caregiver’s health but also the care that is being provided to the patient.^43^ The generic measure, FROM-16, with established measurement properties such as score meanings for ease of interpretation and responsiveness to change^14^ could potentially be used to measure this impact in clinical practice to support affected family members. This could in turn, improve patient outcomes and reduce the financial toll of providing long-term care for the patient. Given that FROM-16 has been successfully mapped to EQ-5D,^16^ the data from routine practice can now allow the inclusion of the economic value of dementia caregiving in the appraisal of health technology assessments.

## CONCLUSIONS

To conclude, this study demonstrates that dementia has a great impact on the QoL of family members/partners of patients. Healthcare professionals and support services can use FROM-16 to have a deeper understanding of the impact of dementia caregiving, identifying those affected the most and providing them with care and support. Such routine FROM-16 data can also be used for economic appraisal of the family burden of dementia as well as family carers' forgotten substantial economic contribution.

## Supplementary Material
