# Supplementary material for: Dementia and Its Profound Impact on Family Members and Partners: A Large UK Cross-Sectional Study
Source: Alzheimer Dis Assoc Disord. 2024 Nov 7;38(4):338–43. doi: 10.1097/WAD.0000000000000647 (PMC11584184; doi:10.1097/WAD.0000000000000647)
Supplement: Supplementary file 1 [file wad-38-338-s001.docx]

**Supplementary material**

**Dementia and its profound impact on family members and partners: a large UK cross-sectional study**

**INDEX: Tables/figures included in the online resource**

Table S1 Comparisons^Ϯ^ of FROM-16 scores based on family member gender

Table S2 FROM-16 scores across different age groups

Table S3a Comparison^Ϯ^ of QoL impact across age groups (18-29, 30-59, 60-79, 80-91 yrs)

| Table S3b Comparison^ϯ^ across age four groups for emotional impact on family members |
| --- |

Table S4 FROM-16 scores across different relationships

Table S5a Comparison^Ϯ^ of QoL impact across relationships (spouse/partners, adult children, parents, siblings and other relatives of people with dementia)

Table S5b Comparison^ϯ^ of impact on QoL across relationships

**Table S1** Comparisons^Ϯ^ of FROM-16 scores based on family member gender

| FROM-16 | Male  (n=132) | Female  (n=578) | P-value |
| --- | --- | --- | --- |
| **Total FROM-16** | 15.71 | 17.88 | 0.001 |
| **FROM-16 Domain** |  |  |  |
| Emotional | 6.71 | 8.11 | < 0.001 |
| Personal and Social | 9.00 | 9.76 | 0.116 |
| **FROM-16 items** |  |  |  |
| Worried | 1.39 | **1.63** | < 0.001 |
| Feeling angry | 0.55 | **0.93** | < 0.001 |
| Feeling Sad | 1.44 | **1.7** | < 0.001 |
| Feeling frustrated | 1.26 | **1.45** | 0.002 |
| Talking about thoughts | 0.70 | **0.9** | 0.006 |
| Difficulty caring | 1.37 | **1.51** | 0.057 |
| Time for self | 1.06 | 1.16 | 0.159 |
| Daily Travel | 0.73 | 0.7 | 0.841 |
| Eating habits | 0.52 | 0.58 | 0.335 |
| Family activities | 1.20 | 1.29 | 0.23 |
| Holiday | 1.20 | 1.23 | 0.747 |
| Sex life | **0.84** | 0.62 | 0.01 |
| Work or study | 0.61 | **0.82** | 0.005 |
| Family relationships | 0.86 | **1.16** | < 0.001 |
| Family expenses | 0.98 | 0.98 | 0.945 |
| Sleep | 0.98 | **1.22** | < 0.001 |

^Ϯ^ Independent Sample t-Test **;** *p-value significant at ≤0.05

Table S2 FROM-16 scores across different age groups

| Family member | FROM-16 | | | |  | | Domain score | | |
| --- | --- | --- | --- | --- | --- | --- | --- | --- | --- |
| (Age group) | Mean(SD) | Median | Range | IQR | | Emotional | | Personal and Social life |  |
| 18-29 (n=16) | 15.06(6.7) | 15.50 | 0-27 | 9.25 | | 7.56 | | 7.50 | |
| 30-59 (n=323) | 17.63 (6.6) | 17.00 | 1-32 | 9 | | 8.13 | | 9.51 | |
| 60-79 (n=345) | 17.50 (6.9) | 17.00 | 1-32 | 10 | | 7.69 | | 9.81 | |
| 80-91 (n=27)) | 16.63 (8.3) | 16.63 | 3-30 | 16 | | 6.89 | | 9.74 | |

Table S3a Comparison^Ϯ^ of QoL impact across age groups (18-29, 30-59, 60-79, 80-91 yrs)

| FROM-16 | | Sum of Squares | df | Mean Square | F | Sig*. |
| --- | --- | --- | --- | --- | --- | --- |
| Emotional domain | Between Groups | 60.958 | 3 | 20.319 | 3.066 | **.027** |
|  | Within Groups | 4685.694 | 707 | 6.628 |  |  |
|  | Total | 4746.653 | 710 |  |  |  |
| Personal and Social Life  domain | Between Groups | 89.882 | 3 | 29.961 | 1.166 | .322 |
|  | Within Groups | 18174.062 | 707 | 25.706 |  |  |
|  | Total | 18263.944 | 710 |  |  |  |
| Total FROM16 Score | Between Groups | 120.950 | 3 | 40.317 | .872 | .455 |
|  | Within Groups | 32678.369 | 707 | 46.221 |  |  |
|  | Total | 32799.319 | 710 |  |  |  |

ϮANOVA; *p-value significant at ≤0.05

| Table S3b Comparison^ϯ^ across four age groups for emotional impact on family members | | | | | | | |
| --- | --- | --- | --- | --- | --- | --- | --- |
| Age Group | Comparison with other Age Group | Mean Difference (I-J) | Std. Error | Sig*. | 95% Confidence Interval | | |
|  |  |  |  |  | Lower Bound | Upper Bound | |
| 18-29 | 30-59 | -.57063 | .65935 | .823 | -2.2686 | 1.1273 | |
|  | 60-79 | -.12736 | .65836 | .997 | -1.8227 | 1.5680 | |
|  | 80-91 | .67361 | .81221 | .841 | -1.4180 | 2.7652 | |
| 30-59 | 18-29 | .57063 | .65935 | .823 | -1.1273 | 2.2686 | |
|  | 60-79 | .44327 | .19932 | .118 | -.0700 | .9566 | |
|  | 80-91 | 1.24424 | .51574 | .076 | -.0839 | 2.5723 | |
| 60-79 | 18-29 | .12736 | .65836 | .997 | -1.5680 | 1.8227 | |
|  | 30-59 | -.44327 | .19932 | .118 | -.9566 | .0700 | |
|  | 80-91 | .80097 | .51447 | .404 | -.5239 | 2.1258 | |
| 80-91 | 18-29 | -.67361 | .81221 | .841 | -2.7652 | 1.4180 | |
|  | 30-59 | -1.24424 | .51574 | .076 | -2.5723 | .0839 | |
|  | 60-79 | -.80097 | .51447 | .404 | -2.1258 | .5239 | |
| ^ϯ^ ANOVA Post-Hoc test; *p-value significant at ≤0.05 | | | | | | |  |

Table S4 FROM-16 scores across different relationships

|  |  | FROM-16 |  |  |
| --- | --- | --- | --- | --- |
| Relationship of family member  to a person with dementia | Mean(SD) | Median | Range | IQR |
| Spouses/partners (n=156) | 19.46(7.3) | 20 | 0-32 | 11.75 |
| Adult children (n=451) | 17.24(6.5) | 16 | 1-32 | 9 |
| Parents (n=13) | 18.31(6.8) | 17 | 6-30 | 9 |
| Siblings (n=22) | 13.55(6.9) | 14 | 3-26 | 10.75 |
| Others (n=69) | 15.65(6.7) | 16 | 1-30 | 11 |

Table S5a Comparison of QoL impact across relationships (spouse/partners, adult children, parents, siblings and other relatives of people with dementia)

| ANOVA | Sum of Squares | df | Mean Square | F | Sig. |
| --- | --- | --- | --- | --- | --- |
| Between Groups | 1215.143 | 4 | 303.786 | 6.791 | .0001 |
| Within Groups | 31584.176 | 706 | 44.737 |  |  |
| Total | 32799.319 | 710 |  |  |  |

Table S5b Comparison^ϯ^ of impact on QoL across relationships

| (I) Family Relationship | (J) Comparison with other relationships | Mean Difference (I-J) | Std. Error | Sig. | 95% Confidence Interval | |
| --- | --- | --- | --- | --- | --- | --- |
|  |  |  |  |  | Lower Bound | Upper Bound |
| **Spouse / Partner** | Son/daughter | 2.218^*^ | .621 | **.003** | .52 | 3.92 |
|  | Parent | 1.147 | 1.931 | .976 | -4.13 | 6.43 |
|  | Sibling | 5.910^*^ | 1.523 | **.001** | 1.74 | 10.08 |
|  | Other | 3.803^*^ | .967 | **.001** | 1.16 | 6.45 |
| **Son/daughter** | Spouse/Partner | -2.218^*^ | .621 | **.003** | -3.92 | -.52 |
|  | Parent | -1.070 | 1.882 | .980 | -6.22 | 4.08 |
|  | Sibling | 3.692 | 1.460 | .086 | -.30 | 7.69 |
|  | Other | 1.585 | .865 | .355 | -.78 | 3.95 |
| **parent** | Spouse/Partner | -1.147 | 1.931 | .976 | -6.43 | 4.13 |
|  | Son/daughter | 1.070 | 1.882 | .980 | -4.08 | 6.22 |
|  | Siblings | 4.762 | 2.340 | .250 | -1.64 | 11.16 |
|  | Other | 2.656 | 2.022 | .683 | -2.88 | 8.19 |
| **Sibling** | Spouse/Partner | -5.910^*^ | 1.523 | **.001** | -10.08 | -1.74 |
|  | Son/daughter | -3.692 | 1.460 | .086 | -7.69 | .30 |
|  | Parent | -4.762 | 2.340 | .250 | -11.16 | 1.64 |
|  | Other | -2.107 | 1.638 | .700 | -6.59 | 2.37 |
| **Other** | Spouse / Partner | -3.803^*^ | .967 | .001 | -6.45 | -1.16 |
|  | Son/daughter | -1.585 | .865 | .355 | -3.95 | .78 |
|  | Parent | -2.656 | 2.022 | .683 | -8.19 | 2.88 |
|  | Sibling | 2.107 | 1.638 | .700 | -2.37 | 6.59 |
| ϯANOVA Post-Hoc Anova test; *The mean difference is significant at 0.05. | | | | | | |
